# A Rare Case of Sphenoid Sinus Hemangioma With Intrasellar and Cavernous Sinus Extension

**DOI:** 10.7759/cureus.61034

**Published:** 2024-05-25

**Authors:** Mikiya Fujii, Kota Yokoyama, Yoji Tanaka, Daisuke Kobayashi, Ukihide Tateishi

**Affiliations:** 1 Department of Diagnostic Radiology and Nuclear Medicine, Tokyo Medical and Dental University, Tokyo, JPN; 2 Department of Neurosurgery, Tokyo Medical and Dental University, Tokyo, JPN; 3 Department of Pathology, Nerima-Hikarigaoka Hospital, Tokyo, JPN; 4 Department of Pathology, Tokyo Medical and Dental University, Tokyo, JPN

**Keywords:** angiography, mri, sphenoid sinus, vascular malformation, hemangioma

## Abstract

Sphenoid sinus hemangiomas are uncommon and pose significant diagnostic challenges due to their rarity and the complex symptoms associated with their critical anatomical location. This report discusses a woman in her 40s who presented with worsening headaches, diplopia, and a sensation of pressure behind her eyes. Diagnostic imaging revealed a lobulated mass in the sphenoid sinus extending into the cavernous sinus and sella, initially mimicking an aggressive neoplastic pathology. However, histopathological examination following endovascular embolization and partial surgical resection confirmed the diagnosis of a cavernous hemangioma. This case highlights the importance of considering hemangiomas in the differential diagnosis of sphenoid sinus masses, especially when patients present with atypical symptoms and imaging shows features such as high vascularity and bone remodeling. The findings emphasize the need for careful diagnostic and therapeutic approaches to effectively manage such cases and differentiate them from more aggressive pathologies.

## Introduction

Hemangiomas of the head and neck, though benign, present unique diagnostic challenges due to their variable manifestations based on location [1-5]. As defined in the WHO's 5th Edition of Head and Neck Tumors, these vascular tumors show a preference for mucosal sites such as the oral cavity and the sinonasal tract [2]. The sphenoid sinus hemangioma, a particularly rare form, exemplifies these challenges due to its critical location and its potential to mimic more aggressive pathologies [6]. Key distinguishing features of hemangiomas include intense radiographic enhancement and the potential for bone remodeling, which are crucial for differentiating them from their malignant counterparts. Although the pathogenesis of hemangiomas is not fully understood, with recent genetic studies suggesting a potential origin from ectopic placental cells in congenital cases [7], this case report underscores the importance of understanding the morphological imaging characteristics of hemangiomas in rare locations. This is essential not only for accurate diagnosis but also for appropriate management to prevent overtreatment and ensure optimal patient outcomes. This report of a sphenoid sinus hemangioma extending into the intrasellar and cavernous sinuses emphasizes the need for meticulous evaluation in such complex cases.

## Case presentation

**Figure 1:**
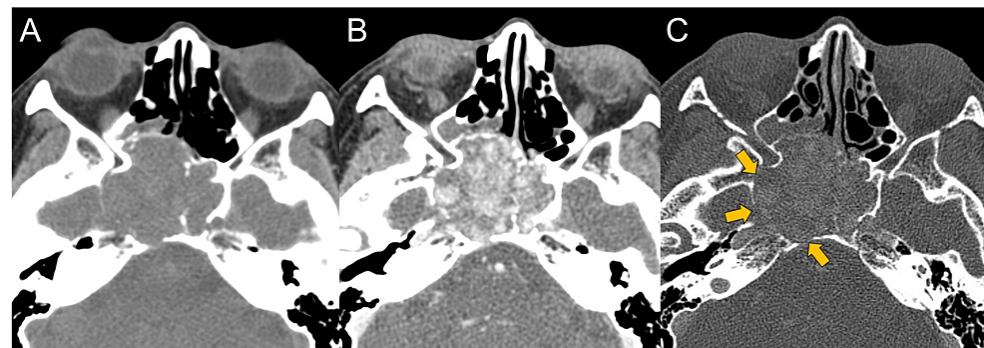
Non-contrast and contrast-enhanced computed tomography at the time of the visit On a computed tomography (CT) scan, the mass shows expansive growth and iso-density on non-contrast CT (A). Contrast-enhanced CT in the arterial dominant phase revealed enhanced vessel-like structures within the lesion (B). There is remodeling rather than destruction of the surrounding bones (C, yellow arrows).

A woman in her 40s presented to our hospital with a history of progressively worsening headaches, diplopia, and a sensation of pressure behind her eyes over the past five months. She also reported an increase in the frequency of epistaxis, which she had experienced occasionally since childhood. A physical examination showed restricted abduction in the right eye. Her medical history included only hyperlipidemia and allergic rhinitis, with no other significant past illnesses or conditions. Non-contrast computed tomography (CT) revealed a well-defined lobulated mass measuring 46×45×37 mm within the sphenoid sinus (Figure 1A), demonstrating expansile growth and bone remodeling (Figure 1C). Contrast-enhanced CT, although not a dynamic study, was taken in the arterial dominant phase and revealed enhancing vessel-like structures within the lesion (Figure 1B).

**Figure 2:**
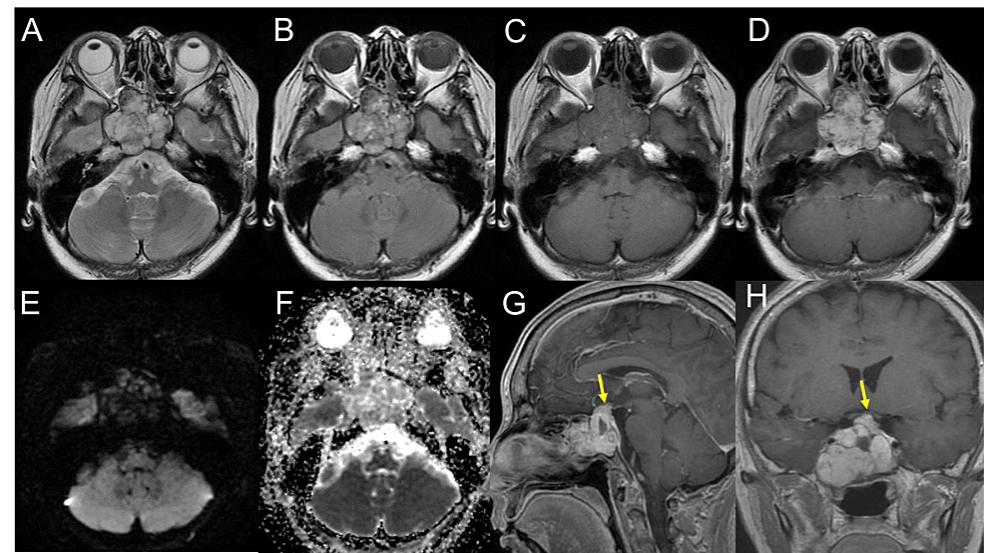
Preoperative magnetic resonance imaging On the magnetic resonance imaging (MRI) scan, the lesion shows predominantly hyperintensity with hypointense, thin internal septal structures on the T2-weighted image (A) and fluid-attenuated inversion recovery (B). The pre- (C) and post-contrast (D) T1-weighted image (T1WI) revealed the avid enhancement of the mass. The lesion did not show restrictions on the diffusion-weighted image (E) or apparent diffusion coefficient map (F). On sagittal (G) and coronal (H) post-contrast T1WI, the lesion was located mainly at the sphenoid sinus, involving the cavernous sinus and the sella. The optic chiasm was compressed by the lesion (G, H, yellow arrow).

Magnetic resonance imaging displayed the mass as predominantly hyperintensity, with hypointense, thin internal septal structures on T2-weighted images (T2WI) (Figure 2A), heterogeneous signals on fluid-attenuated inversion recovery images (Figure 2B), and isointense structures on T1-weighted images (T1WI) (Figure 2C), with avid enhancement following contrast administration (Figure 2D). No diffusion restriction was noted (Figures 2E, 2F). The lesion primarily occupied the sphenoid sinus, extending into the cavernous sinus and the Sella, with noted compression of the optic chiasm (Figures 2G, 2H).

**Figure 3:**
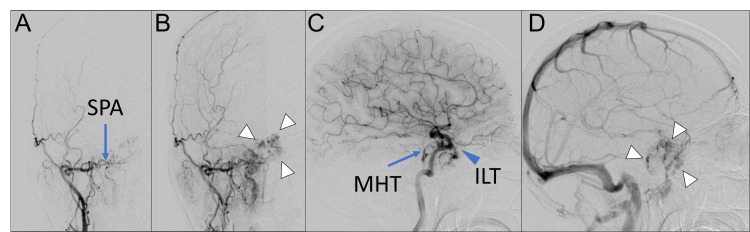
Preoperative angiography of the internal and external carotid arteries An anteroposterior view of the right external carotid angiography (A-B) revealed the vascular pooling of the mass (B, white arrowhead) supplied by the sphenopalatine artery (SPA) (A, blue arrow). On a lateral view of the internal carotid angiography (C-D), the vascular pooling of the mass (D, white arrowhead) supplied by the meningohypophyseal trunk (MHT) (C, blue arrow) and inferolateral trunk (ILT) (C, blue arrowhead) is revealed.

Angiography revealed vascular pooling into the mass from branches of the external carotid artery, including the sphenopalatine artery, as well as branches of the internal carotid artery, such as the inferolateral trunk and meningohypophyseal trunk (Figure 3).

Preoperative multimodality imaging suggested a hypervascular mass in the sphenoid sinus. Despite the high signal intensity on T2WI, the presence of septal structures and low-signal components indicated a heterogeneous signal, suggesting the possibility of fibrous components. Although the patient’s age and gender were not typical for juvenile angiofibroma, they were considered in the differential diagnosis due to the imaging characteristics. A solitary fibrous tumor was also considered based on the T2WI heterogeneity, and paraganglioma was considered due to the hypervascular nature of the mass. Retrospectively, the imaging features were consistent with those reported for hemangiomas, but this was not included in the differential diagnosis preoperatively.

Perioperative endovascular coil embolization of the sphenopalatine artery and combined extended transbasal and endoscopic transnasal surgical approaches were performed, achieving a subtotal excision of the tumor.

**Figure 4:**
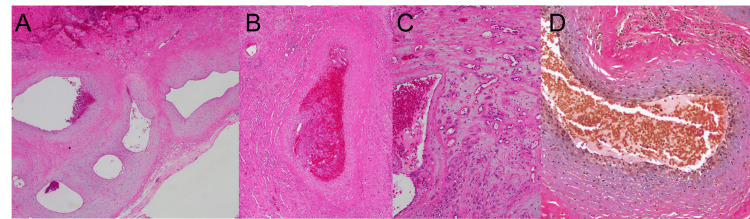
Histopathology of the sphenoid sinus mass Hematoxylin and eosin staining (A-C) and Elastica van Gieson staining (D) of the sphenoid sinus hemangioma tissue specimen. Histopathological examination revealed that the lesion was predominantly composed of large vessels (A). Thrombosis (B) and several capillary vessels (C) are also depicted. No internal elastic lamina was observed on the vessel walls (D).

Histopathological examination predominantly showed large vessels (Figure 4A) with partial luminal occlusion due to thrombosis (Figure 4B). Clusters of capillaries were also noted (Figure 4C). Internal elastic lamina in the vessel walls was absent (Figure 4D), and no tumor cells were observed, which led to a diagnosis of hemangioma.

Postoperatively, the patient's symptoms improved, and she has been under follow-up with imaging. Nine years post-operation, there has been no recurrence of symptoms or lesions.

## Discussion

This case presents a diagnostic challenge involving a sphenoid sinus mass that extended into the Sella and cavernous sinus, accompanied by significant bone remodeling. The imaging and clinical progression initially mimicked those of an aggressive neoplastic lesion. However, the final diagnosis was hemangioma, notable for the absence of tumor cells. Hemangiomas in the head and neck are defined as benign vascular neoplasms in the WHO classification [2], but they are categorized as a vascular malformation according to the latest International Society for the Study of Vascular Anomalies (ISSVA) classification published in 2018, due to the absence of atypical cells, and the term “-oma” is not preferred for non-neoplastic lesions [8,9]. Nevertheless, the mass lesions, so-called hemangioma, may be acceptable in imaging. The incidence rate of hemangiomas is approximately 1/2000-1/10000, with 40% occurring in the head and neck region [5,10]. Among hemangiomas in the head and neck region, 12.5% occur in the sinonasal area, with the most common sites being the nasal septum (40.5%) followed by the posterior nasal cavity (29.7%), while the involvement of the maxillary sinus is less frequent, reported at 8.1% [11]. Reports of sphenoid sinus hemangioma are extremely rare [6], as no sphenoid sinus involvement was documented in the largest study, which examined 37 sinonasal hemangiomas over 20 years at a single center [11]. The growth rate of hemangiomas is relatively slow, displaying an expansive growth pattern with well-circumscribed margins. Sphenoid sinus hemangiomas can present with central nervous system (CNS) symptoms such as headaches, visual impairment, diplopia, and olfactory disturbances, depending on the size and location of the lesion [1,6,12,13]. The present case showed similar CNS symptoms as well, consistent with the rare but documented progression of sphenoid sinus hemangiomas reported in the literature [6,11,12]. On imaging, hemangiomas typically demonstrate hypointense to isointense on T1WI, hyperintense on T2WI, and exhibit strong contrast enhancement, indicative of abundant vascular lumina. Although reports of imaging findings of sphenoid sinus hemangiomas are rare, they often appear slightly heterogeneous internally compared to the other locations, possibly due to bleeding effects [6,11]. Additionally, cases involving bone remodeling and progression into the cavernous sinus have been reported [6,14]. The differential diagnosis for sphenoid sinus hemangioma includes neoplasms such as chordoma, chondrosarcoma, and carcinoma, which commonly occur in this region, as well as juvenile angiofibroma, solitary fibrous tumor, paraganglioma, and metastases from hypervascular tumors such as clear cell renal cell carcinoma, hepatic cell carcinoma, and neuroendocrine tumors, which can also present as hypervascular masses in this location. Although there may be overlap in imaging and clinical findings, imaging features suggesting bone remodeling or high vascularity, as well as symptoms such as epistaxis, are considered helpful in the diagnosis of sphenoid sinus hemangioma. Pathologically, hemangiomas are primarily classified into capillary and cavernous types based on the dominant vessel size [1], and the present case showed the predominance of large artery-like vessels over capillary regions, which supports the diagnosis of a cavernous hemangioma rather than a capillary hemangioma. A broader categorization, such as "hemangioma," may be appropriate.

Treatment for sphenoid sinus hemangioma often requires surgical intervention, particularly when symptomatic. Previous reports have predominantly reported undergoing endoscopic endonasal surgery [1,6,12,13,15]. Recurrence after excision is uncommon, and the prognosis is generally favorable. However, vascular malformations involving the cavernous sinus carry a risk of bleeding, and biopsy or surgery can lead to massive hemorrhage, potentially resulting in fatal outcomes [16]. When hypervascularity is identified on imaging, it is necessary to exercise caution during invasive procedures. Preoperative embolization should be considered to minimize intraoperative bleeding and facilitate a safer surgical intervention [15].

## Conclusions

This case underscores the diagnostic complexities associated with sphenoid sinus hemangiomas, particularly when they mimic more aggressive neoplastic processes. The diagnosis was challenging due to the hemangioma's rare location and its similar presentation to malignant tumors on imaging. However, careful evaluation, including clinical, radiological, and histopathological examination, was crucial in differentiating it from other serious conditions. This case highlights the need for clinicians to consider hemangiomas in the differential diagnosis when encountering expansile lesions of the sphenoid sinus with significant bone remodeling and high vascularity, even when initial symptoms and imaging may suggest malignancy.

The management of sphenoid sinus hemangiomas involves tailored therapeutic strategies to prevent significant morbidity. This case not only adds to the existing literature by documenting a rare occurrence of sphenoid sinus hemangioma but also emphasizes the importance of a thorough diagnostic approach that integrates clinical, radiological, and pathological data to ensure accurate diagnosis and appropriate management.
